# Methodological quality of English-language genetic guidelines on hereditary breast-cancer screening and management: an evaluation using the AGREE instrument

**DOI:** 10.1186/1741-7015-10-143

**Published:** 2012-11-21

**Authors:** Benedetto Simone, Emma De Feo, Nicola Nicolotti, Walter Ricciardi, Stefania Boccia

**Affiliations:** 1 Institute of Hygiene, Università Cattolica del Sacro Cuore, L.go F. Vito 1, 00168 Rome, Italy; 2 IRCCS San Raffaele Pisana, Rome, Italy

**Keywords:** Breast cancer, BRCA1/2, Familial breast/ovarian cancer, Cancer screening, Cancer surveillance

## Abstract

**Background:**

We examined the methodological quality of guidelines on syndromes conferring genetic susceptibility to breast cancer.

**Methods:**

PubMed, EMBASE, and Google were searched for guidelines published up to October 2010. All guidelines in English were included. The Appraisal of Guidelines, Research and Evaluation (AGREE) instrument was used to assess the quality of the guidelines, and their reported evidence base was evaluated.

**Results:**

Thirteen guidelines were deemed eligible: seven had been developed by independent associations, and the other six had national/state endorsements. Four guidelines performed satisfactorily, achieving a score of greater than 50% in all six AGREE domains. Mean ± SD standardized scores for the six AGREE domains were: 90 ± 9% for 'scope and purpose', 51 ± 18% for 'stakeholder involvement', 55 ± 27% for 'rigour of development', 80 ± 11% for 'clarity and presentation', 37 ± 32% for 'applicability', and 47 ± 38% for 'editorial independence'. Ten of the thirteen guidelines were found to be based on research evidence.

**Conclusions:**

Given the ethical implications and the high costs of genetic testing for hereditary breast cancer, guidelines on this topic should provide clear and evidence-based recommendations. Our analysis shows that there is scope for improving many aspects of the methodological quality of current guidelines. The AGREE instrument is a useful tool, and could be used profitably by guidelines developers to improve the quality of recommendations.

## Background

Breast cancer comprises 22.9% of all cancers in women, and an estimated 460,000 deaths from breast cancer occurred worldwide in 2008, representing around 14% of cancer deaths in women. Breast cancer represents a challenge for public health, and in spite of the extremely high incidence rates, secondary prevention is considered to have a major role in decreasing mortality rates and costs. However, this notion has been challenged by a recent Cochrane review [1], reporting that screening reduces breast-cancer mortality by around 15%, which corresponds to an absolute risk reduction of only 0.05%. Nevertheless, regardless of the real effect of screening on the mortality related to sporadic breast cancer, the current scientific evidence supports secondary prevention for individuals at a high genetic risk of developing breast cancer [2].

A considerable proportion of breast cancers presents with genetic recurrence patterns. The two genes most frequently involved in hereditary breast cancer are the tumor suppressor genes *BRCA1 *and *BRCA2*, which are mutated in approximately 25% of hereditary breast cancers and around 5% of all breast cancers. Woman carrying mutations in either *BRCA1 *or *BRCA2 *have an 80 to 90% lifetime risk of developing breast cancer and a 20 to 50% chance of developing ovarian cancer [3]. Thanks to early multimodal screening, breast cancer in people carrying *BRCA1 *or *BRCA2 *mutations can be diagnosed at an early stage, with consequent favorable effects on their survival and quality of life, and also on costs for the health system [2]. Additionally, carriers can benefit from specific tertiary prevention interventions, as the risk of ovarian, contralateral breast cancer, and of other associated carcinomas (such as prostate, pancreas, and colon) is considerable [3]. It is therefore clear that the identification of mutation carriers of *BRCA1/2 *represents a key issue in public health for the potential implementation of specific prevention and management programs, such as intensive risk-adjusted screening, counseling, and prophylactic treatments [2,3].

The probability that an individual is carrier of a *BRCA1 *or *BRCA2 *mutation can be estimated based on the frequency and age of onset of the disease in relatives and on the organs affected (breast, ovary). Several algorithms are available to estimate the risk of being a carrier of the mutations [2,3]. However, genetic testing, however, is the ultimate tool for diagnosis; issues concerning who should be tested and in which context, and the management of test users, are not easily dealt with, and the tests are expensive, and require a great deal of human resources and expertise. There are also ethical and legal issues that need to be considered; genetic information is sensitive, and data protection is necessary. All these issues need to be clearly addressed by valid, reliable, independent, and easily applicable guidelines. The Appraisal of Guidelines, Research, and Evaluation (AGREE) instrument represents a tool for a thorough quality assessment of guidelines [4]. AGREE is a validated tool produced by the PL96-3669 research program funded by the European Union. It has been developed by researchers and policy-makers from several European countries, as well as Canada, the USA, and New Zealand [4]. Over the past few years, AGREE has become a benchmark in both the evaluation of existing guidelines [5-7] and the development of new ones [8,9]. Application of AGREE has shown that the quality of clinical and preventive guidelines is generally poor [10,11], and that some aspects of their quality, such as their applicability and the involvement of stakeholders, are particularly unsatisfactory [11-13]. The instrument has been applied to guidelines produced in virtually every field of clinical practice, focusing on therapies, treatments, and procedures, and was also recently applied to genetic guidelines on colorectal cancer [11].

The aim of this study was to provide a critical evaluation, using the AGREE instrument, of the quality of guidelines focusing on the management of individuals at higher genetic risk of breast cancer.

## Methods

We searched for guidelines published up to October 2010 that aimed to provide recommendations on the genetic screening, surveillance, and management of people who have or are suspected to have a hereditary breast-cancer susceptibility syndrome. The MedLine, EMBASE and Google databases were searched through using the following terms: (Guidelines OR Recommendations) AND Breast AND Cancer AND Screening AND (BRCA$ OR Hereditary). Reference lists of the eligible papers were also searched manually. We included only guidelines published in English that provided explicit recommendations on the management of individuals who had or were at risk of having genetic forms of breast cancer. When more than one set of guidelines was produced by the same professional body, only the most recently issued was considered. All guidelines on breast-cancer screening reporting non-original (that is, referring to other sets of guidelines on the matter of hereditary forms of breast cancer) recommendations were excluded. For each guideline, we specified the target population and objectives. In particular, the target population was defined as the general population or specific subgroups. Recommendations on breast cancer in men were also reported.

Objectives were grouped as follows.

• Assessment of level of risk for breast cancer (low, average, high) of the target population.

• Definition of the criteria of appropriateness for genetic testing.

• Definition of the criteria for empirical diagnosis of susceptibility syndromes.

• Assessment of surveillance options for individuals with a diagnosis or suspicion of susceptibility syndromes.

• Evaluation of options for prophylactic or post-diagnosis treatments.

Three investigators (BS, EDF, NN) appraised all the selected guidelines using the AGREE instrument [4]. AGREE provides criteria to assess the quality of the methods used for developing the guidelines and of their reporting. The instrument consists of 23 key items organized into 6 domains: 'scope and purpose', 'stakeholder involvement', 'rigour of development', 'clarity and presentation', 'applicability' and 'editorial independence'. Each domain is intended to capture a separate dimension of guideline quality. Items were evaluated independently by the three investigators using a four-point scale as indicated by the AGREE instructions (from 4 (strongly agree) down to 1 (strongly disagree)). The summary score of each domain is calculated by summing the scores of all of the individual items present in the domain, and successively by standardizing the total score as a percentage of the maximum possible score for that domain, as suggested by the authors of AGREE (range 0 to 100%). Item scores were discussed by the three appraisers, and large scoring discrepancies (defined as ≤2 points difference in the score assigned by the evaluators to the same item) were resolved by consensus.

According to the AGREE collaboration Group, based on the results for each of the six domains evaluated, a guideline can be 'strongly recommended',' recommended with provisions', or 'not recommended'. The instrument does not provide criteria to formulate the overall assessment on the guideline, leaving it up to the discretion of the evaluator. We considered as satisfactory any guideline that scored at least 50% in all six of the domains as defined by AGREE. Guidelines were further classified based on whether they were developed by independent associations or by national/state-endorsed societies. The Mann-Whitney test was used to compare the median values of each of the 6 domain scores obtained by applying the AGREE instrument to the 17 guidelines, based on the presence or absence of an endorsement.

We also integrated the AGREE instrument by applying an additional system aimed at evaluating whether guidelines could be considered evidence-based. Following a scheme already proposed in the literature [11,14], we defined three criteria for this purpose: the search strategy having been reported in at least one database, the quality of evidence classified, and the strength of recommendations reported.

## Results

### Literature search

**Figure 1:**
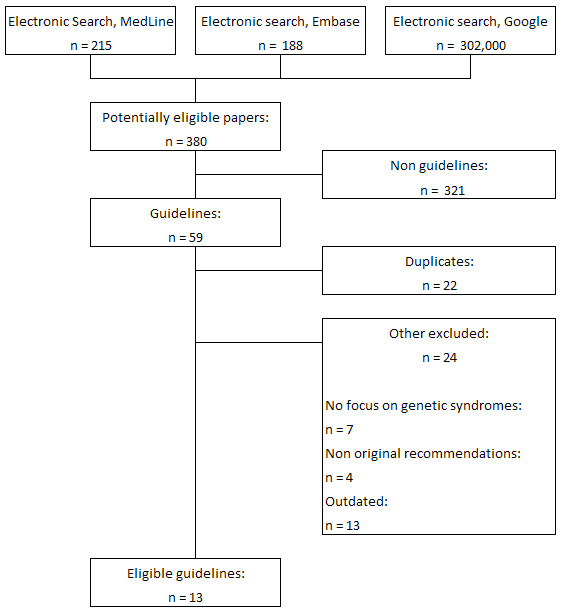
**Flowchart of the guidelines selection process**.

The electronic databases search identified 215 results from MedLine, 188 from EMBASE, and over 302,000 from Google. After a first reading of the titles, any results that were not guidelines were excluded. Duplicates were also excluded, and the application of the inclusion and exclusion criteria (Figure 1) led to the final selection of 13 sets of guidelines (detailed in Table 1) [2,15-27]. All the selected guidelines were developed in English-speaking countries because of the restrictions used in the research (eight from the USA [2,16,17,19-21,25,27], two from the UK [23,24,26], and one each from Canada [22], New Zealand [18] and Singapore [15]). Of the 13 guidelines, 7 were produced by independent professional scientific societies [2,16,17,19-21,27], whereas six were developed with the endorsement of national/state authorities [15,18,22-26] (Table 1).

**Table 1:** Description of the thirteen breast cancer screening guidelines included in the study.

Guidelines (organization), year	Evidence base	Syndromes considered	Target population	Risk assessment	Criteria for genetic risk evaluation	Diagnosis and counselling	Treatment
Breast Cancer Screening and Diagnosis (National Cancer Comprehensive Network), 2010^1 ^[2]	Yes	*BRCA1/2*, CS, LFS	General population	Yes	Yes	Yes	Yes
Health Screening, MOH Clinical Practice Guidelines (Ministry of Health, Singapore), 2010^2 ^[15]	Yes	*BRCA1/2 *	General population	No	No	No	Yes
Hereditary Breast and Ovarian Cancer Syndrome (American College of Obstetricians and Gynecologists), 2009^1 ^[16]	No	*BRCA1/2 *	General population	Yes	Yes	Yes	Yes
Diagnosis of Breast Disease (Institute for Clinical Systems Improvement), 2008^1 ^[17]	Yes	*BRCA1/2*, CS, LFS, others^3^	General population	No	Yes	Yes	Yes
Management of Early Breast Cancer (New Zealand Guidelines Group), 2008^2 ^[18]	Yes	*BRCA1/2*, CS, LFS other ^3^	General population	Yes	Yes	Yes	Yes
Guidelines for Breast Screening with MRI as an Adjunct to Mammography (American Cancer Society), 2007^1 ^[19]	Yes	*BRCA1/2*, CS, LFS	General population	Yes	Yes	No	Yes
Risk Assessment and Genetic Counseling for Hereditary Breast and Ovarian Cancer: Recommendations (National Society of Genetic Counselors), 2007^1 ^[20]	No	*BRCA1/2*, CS, LFS, other^3^	General Population	Yes	Yes	Yes	Yes
Adult Preventive Health Care: Cancer Screening (University of Michigan), 2007^1 ^[21]	No	*BRCA1*/2	General population	No	No	No	Yes
The Early Detection of Breast Cancer (Towards Optimized Practice Alberta), 2007^2 ^[22]	Yes	*BRCA1/2*	General population	Yes	Yes	Yes	Yes
Familial breast cancer + Update (NHS), 2006^2 ^[23,24]	Yes	*BRCA1/2*, LFS	General population	Yes	Yes	Yes	Yes
Genetic Risk Assessment and BRCA Mutation Testing for Breast and Ovarian Cancer Susceptibility: Recommendation Statement (U.S. Preventive Services Task Force), 2004^2 ^[25]	Yes	*BRCA1/2*	General population	Yes	Yes	Yes	No
Management of Breast Cancer in Women (Scottish Intercollegiate Guidelines Group), 2004^2 ^[26]	Yes	*BRCA1/2*	General population	Yes	Yes	Yes	Yes
Guidelines for Breast Cancer Screening (American Cancer Society), 2003^1 ^[27]	Yes	*BRCA1/2*, CS, LFS	General population	Yes	Yes	Yes	Yes

Abbreviations: CS, Cowden syndrome; LFS, Li-Fraumeni syndrome

^1^Independent body/no endorsement.

^2^National/state endorsement.

^3^Other syndromes: Bannayan-Riley-Ruvalcaba syndrome, Peutz-Jeghers syndrome, hereditary diffuse gastric cancer and ataxia telangectasia.

### Target population and objectives of guidelines

The guidelines analyzed are relatively homogeneous in terms of target populations: they all begin by focusing on the general population and then provide specific recommendations on patients with high-risk syndromes. Regarding the objectives, surveillance recommendations are provided by all the guidelines, but not all give indications about how to perform a risk assessment [15,17,21], criteria of appropriateness for genetic testing [15,21], the definition of empirical diagnostic criteria of susceptibility syndromes [15,19,21] or the available treatment options [25]. Apart from the BRCA1/2 syndromes, most guidelines also provide recommendations on, or at least mention, less common syndromes such as Li-Fraumeni, Peutz-Jeghers, and Cowden syndromes (Table 1). Although the main recommendations are focused on women, all the guidelines provide at least some recommendations on syndromic breast cancer in men.

### Appraisal of guidelines

Based on the criteria defined in the methods section, 10 (77%) of the 13 guidelines are evidence-based [2,15,17-19,22-27] (table 1), and apart from the 3 exceptions [16,20,21], all guidelines stated, either in the text or in a clearly specified link, the methods used in the literature search, the quality of the evidence, and the strength of recommendations reported.

Application of the AGREE instrument produced six standardized scores for each guideline, pertaining to the specific domain (Table 2). We deemed satisfactory the guidelines produced by the Institute for Clinical Systems Improvement (ICSI)[17], The New Zealand Guidelines Group (NZGG) [18], the UK National Health System (NHS) [23,24] and the Scottish Intercollegiate Guidelines Network (SIGN) [26], which all had a score of at least 50% in each of the six domains. All the other guidelines scored below 50% in at least one domain. The lowest scores were assigned to the Guidelines produced by the Ministry of Health of Singapore [15], with five of the six domains scoring below 50%. Two more guidelines [16,22] also achieved scores of below 50% in four of the six domains (Table 2).

**Table 2:** Standardized scores (%) on the Appraisal of Guidelines, Research and Evaluation (AGREE) instrument assigned to the 13 guidelines.

	Domain number (name)					
	**1 (Scope and purpose)**	**2 (Stakeholder involvement)**	**3 (Rigour of development)**	**4 (Clarity and presentation)**	**5 (Applicability)**	**6 (Editorial independence)**

National Cancer Comprehensive Network^1 ^[2]	92.6	63.9	50.8	83.3	7.4	100.0

Ministry of Health, Singapore^2 ^[15]	7.4	47.2	12.7	69.4	25.9	0.0

American College of Obstetricians and Gynecologists^1 ^[16]	100.0	44.4	33.3	77.8	18.5	0.0

Insitute for Clinical Systems Improvement^1 ^[17]	74.1	50.0	52.4	80.6	55.6	55.6

New Zealand Guidelines Group^2 ^[18]	81.5	75.0	81.0	100.0	85.2	100.0

American Cancer Society (MRI)^1 ^[19]	96.3	52.8	65.1	86.1	44.4	0.0

National Society of Genetic Counselors^1 ^[20]	100.0	41.7	69.8	72.2	44.4	50.0

University of Michigan^1 ^[21]	92.6	8.3	39.7	80.6	0.0	88.9

Towards Optimized Practice Alberta^2 ^[22]	74.1	33.3	7.9	69.4	18.5	0.0

National Health System^2 ^[23,24]	88.9	75.0	87.3	88.9	77.8	50.0

U.S. Preventive Services Task Force^2 ^[25]	96.3	72.2	69.8	86.1	3.7	50.0

Scottish Intercollegiate Guidelines Group^2 ^[26]	96.3	50.0	96.8	86.1	88.9	72.2

American Cancer Society^1 ^[27]	92.6	47.2	52.4	55.6	11.1	50.0

^1^Independent body/no endorsement,.

^2^National/state endorsement,

As shown in Table 2, the highest score (100%) for domain 1 (scope and purpose) was given to the guidelines of the American College of Obstetricians and Gynecologists (ACOG) [16] and the National Society of Genetic Counselors [20], whereas the lowest score (7%) was assigned to the Singapore guideline [15]. Scores for domain 2 (stakeholder involvement) were generally very low, ranging from 75% (NZGG [18] and NHS [23,24]) to 8% (University of Michigan [21]). The SIGN guidelines [26] gained the highest score (97%) for domain 3 (rigour of development), whereas the lowest (8%) was assigned to the guidelines from Towards Optimized Practice Alberta [22]. The highest score (100%) assigned to domain 4 (clarity and presentation) was achieved by the NZGG [18], and the lowest (56%) by the American Cancer Society [27]. SIGN [26] had the best score (89%) in domain 5 (applicability), whereas the University of Michigan had the worst (0%) [21]. Finally, the top scores (100%) for domain 6 (editorial independence) were obtained by the National Cancer Comprehensive Network [2] and the NZGG [17], whereas four guidelines [15,16,19,22] scored 0% in this domain for not being explicit on conflicts of interest and on independence statements from funding bodies.

Table 3 details the overall mean scores for all the 23 items included in the 6 domains, and the overall mean standardized scores for each of the 6 domains from the 13 guidelines evaluated. The highest score was obtained for domain 1 (scope and purpose) with a value of 90 ± 9%, and domain 4 (clarity and presentation) with 80 ± 11%, whereas the lowest scores were for domain 5 (applicability) with 37 ± 32%, and domain 6 (editorial independence) with 47 ± 38%. Domains 2 (stakeholder involvement) and 3 (rigour of development) scored overall 51 ± 18% and 55 ± 27%, respectively (Table 3).

**Table 3:** Mean scores for the 23 items and overall standardized scores for each domain from the 13 guidelines evaluated assessed with AGREE.

Domain	Item	Mean score (range)
1 (Scope and purpose)	• The overall objective(s) of the guideline is (are) specifically described	3.7 (2.3 to 4.0)
	• The clinical question(s) covered by the guideline is(are) specifically described	3.6 (2.7 to 4.0)
	• The patients to whom the guideline is meant to apply are specifically described	3.7 (3.3 to 4.0)
	Overall standardized score, %	89.5 (66.7 to 100.0)

2 (Stakeholder involvement)	• The guideline development group includes individuals from all the relevant professional groups	3.3 (1.3 to 4.0)
	• The patients' views and preferences have been sought	2.7 (1.3 to 4.0)
	• The target users of the guideline are clearly defined	3.0 (1.0 to 4.0)
	• The guideline has been piloted among end users	1.1 (1.0 to 1.7)
	Overall standardized score, %)	50.9 (8.3 to 75.0)

3 (Rigour of development)	• Systematic methods were used to search for evidence	2.6 (1.0 to 4.0)
	• The criteria for selecting the evidence are clearly described	2.8 (1.0 to 4.0)
	• The methods used for formulating the recommendations are clearly described	3.0 (1.0 to 4.0)
	• The health benefits, side effects, and risks have been considered in formulating the recommendations	3.1 (1.7 to 4.0)
	• There is an explicit link between the recommendations and the supporting evidence	3.1 (1.3 to 4.0)
	• The guideline has been externally reviewed by experts before its publication	2.3 (1.0 to 4.0)
	• A procedure for updating the guideline is provided	1.7 (1.0 to 4.0)
	Overall standardized score, %	55.5 (7.9 to 96.8)

4 (Clarity and presentation)	• The recommendations are specific and unambiguous	3.4 (2.3 to 4.0)
	• The different options for management of the condition are clearly presented	3.4 (2.3 to 4.0)
	• Key recommendations are easily identifiable	3.7 (2.7 to 4.0)
	• The guideline is supported with tools for application	3.0 (1.0 to 4.0)
	Overall standardized score, %	79.7 (55.6 to 100.0)

5 (Applicability)	• The potential organizational barriers in applying the recommendations have been discussed	2.2 (1.0 to 4.0)
	• The potential cost implications of applying the recommendations have been considered	2.4 (1.0 to 4.0)
	• The guideline presents key review criteria for monitoring and/or audit purposes	1.7 (1.0 to 3.7)
	• Overall standardized score, %	37.0 (0.0 to 88.9)

6 (Editorial independence)	• The guideline is editorially independent from the funding body	2.2 (1.0 to 4.0)
	• Conflicts of interest of guideline development members have been recorded	2.6 (1.0 to 4.0)
	Overall standardized score, %	47.4 (0.0 to 100.0)

Comparison between endorsed and non-endorsed guidelines showed that the former performed better in five of the six domains, although no statistical significance was attained for any domain.

## Discussion

Genetic forms of breast cancer are an issue for public health. Women with a family history of breast cancer, and especially women with genetically known forms of susceptibility, can benefit from appropriate prevention and treatment interventions. Outcomes for breast cancer are strongly associated with the stage and degree of disease progression at the time of diagnosis, and this also holds true for genetically determined forms. Because effective screening surveillance and adequate preventive measures are proven to have a dramatic effect on the survival and the quality of life of individuals with inherited breast-cancer syndromes [1,3], specific recommendations to define high-risk individuals and appropriate screening protocols should be provided. It is essential that, given the ethical implications of genetic testing, and also in consideration of the high costs related to their administration, guidelines should provide very clear and evidence-based recommendations on who should be tested, based on their personal and family history and on clinical criteria.

In this study, we aim to evaluate the quality of methodology of guidelines dealing with the issue of genetic testing for hereditary breast cancer, using the AGREE instrument. The application of AGREE allows evaluation of various aspects of guidelines quality: 'scope and purpose', taking into account whether the objectives, the clinical questions, and the target population are properly specified; 'stakeholder involvement', assessing which professional groups have been involved in the guideline development, and whether patients' views and preferences have been sought; 'rigour of development', with a list of key items focusing on the methods used by the developers, starting from the literature search up to the external review of the recommendations; 'clarity and presentation', focusing on how easily the user is able to find the key recommendations and the possible alternatives in the guideline; 'applicability', with three key items assessing how organizational barriers, potential cost implications, and patient monitoring/audit have been discussed; and 'editorial independence', assessing independence statements and records of potential conflicts of interest of the guideline developers.

The evaluation of the quality of the evidence which the guidelines build upon was beyond our objectives. We focused on the methods used in the course of development of the guidelines, which is the purpose of the AGREE instrument, based on the rationale that high methodological quality is fundamental in terms of credibility, reproducibility and transparency of guidelines. Furthermore, in the case of genetic susceptibility syndromes for breast cancer, as of today, there is a limited body of evidence focusing on the best screening and management options.

All the guidelines considered in this review are based on the same studies, therefore the recommendations necessarily converge. The recommendations on the topic given by the guidelines analyzed are as follows.

• All individuals at high risk (individuals from known high-risk families, or with high scores on the BRCAPRO [28] or BOADICEA [29] programs, or deemed at high risk based on clinical judgment) should be offered referral for information on genetic testing.

• Counseling from training personnel should be always available.

• If a mutation is identified in one individual from a high-risk family, predictive testing should then be offered to all adult at-risk family members.

• Known carriers of a *BRCA1 *or *BRCA2 *gene mutation should be offered counseling and the option of prophylactic mastectomy, and prophylactic salpingo-oophorectomy should also be discussed.

• Individualized screening strategies for known carriers of *BRCA1 *or *BRCA2 *gene mutations should be considered, such as earlier screening, shorter intervals between screens, and possibly annual MRI surveillance.

The most important difference between guidelines, however, and we believe it to be noteworthy, is how the different developers used the same evidence to produce the guidelines. The application of AGREE detected some major flaws in the development of the 13 guidelines on the topic, as some of the aspects investigated by AGREE were not included in these guidelines. With very few exceptions, the 13 guidelines all performed poorly with regard to 'stakeholder involvement' (domain 2) and 'editorial independence' (domain 6). Regarding stakeholder involvement, target users of the guideline (general practitioners, gynecologists, oncologists) remained generally undefined (key item 6), patient representatives were seldom involved (key item 5) in guideline development, and most guidelines were not piloted among end users (key item 7). Regarding editorial independence, explicit statements of independence from funding bodies (key item 22) were often not clearly stated, and did not allow the identification of possible conflicts of interest. The application of AGREE also showed that the methodological quality of the guidelines was suboptimal in terms of 'rigour of development' (domain 3) and 'applicability' (domain 5). Most guidelines lacked explicit statements on the criteria for selecting the evidence (key item 9), on whether they were externally reviewed before publication (key item 13), and on procedures for their update (key item 14). Generally speaking, the AGREE instrument gave high scores for domains 1 (scope and purpose) and 4 (clarity and presentation), even though not all guidelines received fully positive evaluations.

Although there was a good degree of convergence between guidelines in terms of recommendations provided, our study does have implications for clinical practice as well. As mentioned above, the AGREE instrument provides six independent scores for six corresponding aspects of the guidelines; clinicians would be interested primarily in the 'applicability' domain. It is fundamental that recommendations are not only rigorous in method but also feasible when applied to a specific clinical setting. In this sense, we recommend clinicians should rely preferentially on the guidelines that performed better with regards to the 'applicability' domain [18,23,24,26], as those guidelines gave more consideration to issues related to overcoming possible organizational barriers when applying the recommendation (key item 19), and to presenting criteria for monitoring and audit purposes (key item 20).

By applying the AGREE instrument to the 13 guidelines on genetic testing for breast cancer, we found that guidelines developed by the ICSI [17], the NZGG [18], the SIGN [26] and the NHS [23,24] scored above 50% in all six domains, with the NZGG [18], who acknowledged the adoption of AGREE in the guideline development, scoring above 70% in all domains. As for the other guidelines, two [2,25] yielded poor scores (below 50%) in one of the six domains, three [19,20,27] in two of the six domains, one [21] in three of the six domains, two [16,22] in four of the six domains, and one [15] in five of the six domains.

The guidelines produced by societies with an official endorsement tended to perform better with regard to all six the AGREE domains, however, a significant difference was not detected, probably due to the small sample size.

## Conclusions

The high number of guidelines with low methodological quality in the literature on genetic testing for hereditary breast cancer prompted us to evaluate their methodological quality scientifically. We also provided an insight on important factors that have been missed out of some guidelines, and which, in our opinion, should be considered. The whole objective of using the AGREE instrument is to provide a common ground on rigor and transparency of guideline development, and to suggest how to improve on the existing guidelines [4]. In this sense, the most self-explanatory example is that of conflicts of interest; the AGREE instrument recommends that guidelines always report explicitly whether conflicts exist or not. The absence of an explicit statement does not necessarily mean that a conflict of interest exists, but rather that providing such a statement was not a standard procedure in the development of the guidelines. We would recommend that all future guidelines should always state explicitly that conflicts of interest do or do not exist.

It is noteworthy that the results reported here are very similar to those reported for guidelines focusing on genetic forms of colorectal cancer [11]. Although this study and the previous study do not cover the whole subject of genetic-testing guidelines, they certainly corroborate each other in the notion that there is much to be achieved and improved in terms of methodology and quality where genetic tests are concerned.

## Abbreviations

ACOG: American College of Obstetricians and Gynecologists; AGREE: Appraisal of Guidelines, Research and Evaluation; ICSI: Institute for Clinical Systems Improvement; NHS: National Health System; NZGG: New Zealand Guidelines Group; SIGN: Scottish Intercollegiate Guidelines Network.

## Competing interests

The authors declare that they have no competing interests.

## Authors' contributions

BS drafted the manuscript, participated in the design of the study, and performed the statistical analysis. NN and EDF contributed to the systematic review of literature and to the evaluation of the guidelines. WR participated in the design and the conception of the study. SB conceived of the study, coordinated the project, and participated in the design of the study. All authors read and approved the final manuscript.

## Pre-publication history

The pre-publication history for this paper can be accessed here:

http://www.biomedcentral.com/1741-7015/10/143/prepub
